# Antibacterial Activity of Clove Essential Oil (*Syzygium aromaticum*) Against Oxacillin-Resistant *Staphylococcus pseudintermedius* Isolated from Dogs with Otitis Externa

**DOI:** 10.3390/pathogens14070709

**Published:** 2025-07-17

**Authors:** Vanessa Danielle de Freitas, Edison Lorran Jerdlicka Coelho, Janaina Marcela Assunção Rosa Moreira, Valéria Dutra, Valéria Régia Franco Sousa, Arleana do Bom Parto Ferreira de Almeida

**Affiliations:** 1 Postgraduate Program in Veterinary Sciences, Federal University of Mato Grosso (UFMT), Cuiabá 78060-900, Mato Grosso, Brazil; vanessadanielle@hotmail.com (V.D.d.F.); edison.lorran@gmail.com (E.L.J.C.); janarosavet@gmail.com (J.M.A.R.M.); 2 College of Veterinary Medicine (FAVET), Federal University of Mato Grosso (UFMT), Cuiabá 78060-900, Mato Grosso, Brazil; valeriadutra.dutra@gmail.com (V.D.); arleana.almeida@ufmt.br (A.d.B.P.F.d.A.)

**Keywords:** otitis, multidrug resistance, oxacillin, dogs, essential oils

## Abstract

Infections caused by oxacillin-resistant *Staphylococcus pseudintermedius* are increasingly common in veterinary medicine. The indiscriminate use of antibiotics by pet owners worsens this problem, reducing treatment efficacy and creating the need for alternative therapies. This study aimed to evaluate the inhibitory effect of clove essential oil (*Syzygium aromaticum*) on both oxacillin-resistant and susceptible *S. pseudintermedius*. Thirty-five isolates from dogs with otitis externa were analyzed. The bacteria were identified by phenotypic tests and tested for susceptibility to 22 antibiotics using disk diffusion. Resistance genes (*mecA* and *blaZ*) were detected using conventional PCR. Among the isolates, 34.28% (12/35) were positive for *mecA*, and 97.14% (34/35) for *blaZ*. The essential oil’s efficacy was assessed using broth microdilution to determine its minimum inhibitory concentration (MIC). Clove oil showed an average MIC and minimum bactericidal concentration (MBC) of 6.4 mg/mL, inhibiting both resistant and susceptible isolates. In conclusion, clove essential oil demonstrated in vitro antimicrobial activity against *S. pseudintermedius*.

## 1. Introduction

*S. pseudintermedius*, previously identified as *Staphylococcus intermedius*, is a Gram-positive, facultative anaerobic, beta-hemolytic bacterium belonging to the coagulase-positive *Staphylococcus* species. It is part of the *S. intermedius* group (SIG), which includes *S. pseudintermedius*, *S. intermedius*, and *S. delphini*, phenotypically similar species that are difficult to differentiate [1,2]. They colonize the normal microbiota of dogs and, under physiological conditions, do not cause disease. However, they can act as opportunistic pathogens, leading to infections in immunocompromised hosts or under certain predisposing conditions. *S. pseudintermedius* is the most frequently isolated bacterial agent in cases of otitis externa, pyoderma, and skin wounds, and may also be associated with other clinical conditions such as urinary tract and surgical site infections [2,3,4].

Although humans are not permanent hosts of *S. pseudintermedius*, they may temporarily acquire the bacterium through direct contact with infected dogs. Thus, the transmission of this microorganism from dogs to humans poses a public health risk. Additionally, individuals colonized by *S. pseudintermedius* may act as carriers, facilitating the spread of the bacterium among different animals, which is also a concerning factor [5,6,7,8,9,10].

In the past, *S. pseudintermedius* isolates were susceptible to β-lactam antibiotics; however, since 2006, methicillin resistance in *S. pseudintermedius* (MRSP) has emerged as an animal health concern. Since then, an increasing number of cases have been reported, which is alarming as it limits therapeutic options [11,12,13].

MRSP occurs through two main mechanisms. The first involves the production of the β-lactamase enzyme, encoded by the *blaZ* gene, which inactivates β-lactam antibiotics. The second is related to the PBP2a protein, encoded by the *mecA* gene, which reduces the bacterium’s affinity for these antimicrobials. Additionally, MRSP harbors resistance genes to multiple classes of antibiotics, making treatment particularly challenging [14,15,16,17].

The increasing resistance of many bacteria to commonly used antimicrobials has prompted studies aimed at identifying safe and effective alternatives for the treatment of persistent bacterial infections [13]. Given the growing resistance of *S. pseudintermedius* to conventional antimicrobials, the search for alternative therapeutic options has become essential. Among these alternatives, essential oils (EOs) have been widely studied for their antimicrobial properties and potential clinical applications, emerging as an alternative to conventional antimicrobials, as they exhibit biological activity against both Gram-positive and Gram-negative bacteria, as well as effective action against fungi, protozoa, and viruses [18,19,20].

Essential oils are complex mixtures of volatile compounds, primarily obtained through steam distillation, and are mainly composed of mono- and sesquiterpenes in the form of hydrocarbons or their oxygenated derivatives [21].

Clove, belonging to the Myrtaceae family and currently recognized by the scientific name *Syzygium aromaticum*, is a tree species with an elongated crown that can reach an average height of 8–10 m. Regarding its therapeutic applications, authors attribute its pharmaceutical use primarily to eugenol (phenylpropanoid derivative), the most abundant component (up to 88% of the essential oil) [22,23]. Studies have reported antiviral, aphrodisiac, antioxidant, anesthetic, anti-inflammatory, antimicrobial, insecticidal, antidiabetic, and antitumor properties [20]. Studies have observed good antimicrobial activity of clove essential oil (EO) against *S. aureus* and *Escherichia coli* [20,24,25]. Other studies have reported activity against *S. aureus*, *E. coli*, *Salmonella* spp., *Listeria monocytogenes*, *Pseudomonas aeruginosa*, *Klebsiella pneumoniae*, and *Acinetobacter baumannii* [26,27,28]. According to Silvestre et al. [29], the EO exhibits stronger inhibitory effects on Gram-positive bacteria.

Canine otitis externa is one of the main reasons for veterinary consultations, and due to the indiscriminate empirical use of antibiotics, there has been an increasing development of resistance among the causative agents of this disease [4]. As previously mentioned, *S. pseudintermedius* is the most commonly isolated bacterium in dogs with otitis and is currently associated with cases of MRSP. This represents an even greater challenge for antimicrobial therapy in veterinary medicine due to limited treatment options and the pressure to avoid using antimicrobials that are critical for treating serious infections in humans [4,8]. Thus, the objective of this study was to investigate the potential antibacterial effect of clove essential oil (*S. aromaticum*) against methicillin-resistant *S. pseudintermedius* from canine otitis externa, as well as to characterize, evaluate, and expand knowledge on the bacterial resistance profile and the presence of genes associated with oxacillin resistance in *S. pseudintermedius* isolates.

## 2. Materials and Methods

### 2.1. Isolation and Identification

For this study, 35 *S. pseudintermedius* isolates were used, collected from 30 dogs diagnosed with bacterial otitis externa and treated at the Dermatology department of the Veterinary Hospital of UFMT (Federal University of Mato Grosso, Cuiabá, Brazil). Among the 30 dogs, unilateral otic isolates were obtained from 25 dogs, and bilateral isolates from 5 dogs, totaling 35 isolates. Samples were collected using sterile swabs and sent to the Microbiology Laboratory of HOVET-UFMT (Veterinary Hospital of Federal University of Mato Grosso, Cuiabá, Brazil) for bacterial isolation.

The samples were then plated on 5% sheep blood agar and incubated aerobically at 37 °C for 24 h. The isolates were phenotypically identified according to Quinn et al. [30], based on colony morphology, Gram staining, and catalase test, as *Staphylococcus* spp. After phenotypic identification, isolates classified as *Staphylococcus* spp. were stored in Luria-Bertani broth with glycerol, in triplicate, and kept at −80 °C in an ultra-freezer until all *S. pseudintermedius* isolates were genetically identified, after which they were subjected to antimicrobial susceptibility testing.

For species identification of *S. pseudintermedius*, all isolates classified as *Staphylococcus* spp. underwent DNA extraction using the phenol/chloroform method [31]. Species-specific identification was performed by PCR, employing the oligonucleotide primers 5′-TRGGCAGTAGGATTCGTTAA-3′ and 5′-CTTTTGTGCTYCMTTTTGG-3′, which amplify an approximately 926 bp fragment of the *pse* gene, specific for *S. pseudintermedius* species [32].

Amplifications were performed in a final volume of 25 μL, containing 10 ng of DNA, 1 U of Taq DNA polymerase (Invitrogen^®^, Thermo Fisher Scientific, Waltham, MA, USA), 0.2 mM of dNTPs, 2.5 mM of MgCl_2_, 1× PCR buffer 10× (200 mM of Tris-HCl pH 8.4, 500 mM of KCl), and 20 pmol of each oligonucleotide. A strain that had been sequenced and matched with 100% identity to *S. pseudintermedius* NCTC5561 (GenBank accession number LRI34267.1) was used as a positive control, while a DNA-free reaction was used as a negative control. The reaction conditions were an initial denaturation at 95 °C for 2 min, followed by 35 cycles of 95 °C for 30 s, 56 °C for 30 s, and 72 °C for 30 s, and a final extension phase at 72 °C for 2 min. Amplification products were separated by electrophoresis on a 1.5% agarose gel stained with GelRed (Biotium^®^, Fremont, CA, USA) and visualized using the ChemiDoc™ XRS system with Image Lab™ software, version 6.1, alongside a 100 bp DNA Ladder™ molecular weight marker (Fermentas^®^, Thermo Fisher Scientific, Waltham, MA, USA).

### 2.2. Antimicrobial Susceptibility Testing

Isolates identified as *S. pseudintermedius* were removed from the ultra-freezer and subcultured on 5% sheep blood agar to obtain pure cultures and subsequently subjected to antimicrobial susceptibility testing using the disk diffusion method on agar plates, following the standards established by the VET01-S2 [33] and M100-S25 [34] manuals. Bacterial suspensions were then prepared in sterile saline solution, adjusted to a turbidity equivalent to the 0.5 McFarland standard. Using a sterile swab, the suspensions were evenly inoculated over the entire surface of the Mueller–Hinton agar to ensure uniform distribution. Antibiotic-impregnated paper disks were placed onto the agar surface using sterile forceps. The plates were incubated at 37 °C for 18–24 h. After incubation, the diameters of the inhibition zones were measured in millimeters and interpreted. The antimicrobials tested for staphylococci [34,35] were as follows: ampicillin 10 μg (Cecon^®^, São Paulo, Brazil), penicillin G 10 IU (Cecon^®^, São Paulo, Brazil), oxacillin 1 μg (DME^®^, São Paulo, Brazil), cefoxitin 30 μg (Cefar^®^, São Paulo, Brazil), doxycycline 30 μg (Cecon^®^, São Paulo, Brazil), enrofloxacin 5 μg (Cefar^®^, São Paulo, Brazil), amoxicillin/clavulanic acid 30 μg (Cefar^®^, São Paulo, Brazil), gentamicin 10 μg (Cefar^®^, São Paulo, Brazil), neomycin 30 μg (Cecon^®^, São Paulo, Brazil), amikacin 30 μg (Cecon^®^, São Paulo, Brazil), tobramycin 10 μg (Cecon^®^, São Paulo, Brazil), erythromycin 15 μg (Cefar^®^, São Paulo, Brazil), imipenem 10 μg (Cecon^®^, São Paulo, Brazil), polymyxin B 300 IU (Cefar^®^, São Paulo, Brazil), ciprofloxacin 5 μg (Cecon^®^, São Paulo, Brazil), marbofloxacin 5 μg (Cefar^®^, São Paulo, Brazil), cephalexin 30 μg (Cefar^®^, São Paulo, Brazil), sulfamethoxazole/trimethoprim 25 μg (Cefar^®^, São Paulo, Brazil), clindamycin 2 μg (Cefar^®^, São Paulo, Brazil), rifamycin 30 μg (Cecon^®^, São Paulo, Brazil), chloramphenicol 30 μg (Cefar^®^, São Paulo, Brazil), and vancomycin 30 μg (Cefar^®^, São Paulo, Brazil). Isolates were classified as susceptible, intermediate, or resistant based on the measurement of inhibition zone diameters using a specific ruler for antibiogram reading and comparison with values provided in the appropriate tables, according to the disk manufacturer.

Multidrug-resistant isolates were identified using the Multiple Antimicrobial Resistance (MAR) index, calculated as the number of antimicrobials to which the isolate is resistant divided by the total number of antimicrobials tested, where indices ≥ 0.2 indicate multidrug resistance. Another index evaluated was the Multiple-Class Antimicrobial Resistance (MCAR) index, calculated as the ratio between the number of antimicrobial classes to which resistance was observed (at least one drug per class) and the total number of classes tested [36]. Strains exhibiting resistance to at least three antimicrobial classes (MCAR ≥ 0.25) were considered multidrug-resistant [37].

### 2.3. Genotypic Detection of mecA and blaZ

The presence of the *mecA* and *blaZ* genes was detected by conventional PCR in the 35 *S. pseudintermedius* samples. Amplification of the *mecA* gene was performed using the oligonucleotides 5′-CCTAGTAAAGCTCCGGAA-3′ and 5′-CTAGTCCATTCGGTCCA-3′, which amplify a 314 bp fragment [38]. Amplification of the *blaZ* gene was performed using the oligonucleotides 5′-ACTTCAACACCTGCTGCTTTC-3′ and 5′-TGACCACTTTTATCAGCAACC-3′, which amplify a 173 bp fragment [39]. The samples were placed in the thermocycler (Applied Biosystems^®^, Thermo Fisher Scientific, Waltham, Massachusetts, USA) under the following conditions: initial denaturation at 95 °C for 5 min, followed by 30 amplification cycles of 95 °C for 30 s, annealing at 56 °C for 30 s, and extension at 72 °C for 30 s, concluding with a final extension step at 72 °C for 4 min.

The PCR amplification products were stained with Gel Red (Biotium^®^, Fremont, CA, USA) and separated by electrophoresis on a 2% agarose gel, then visualized using the ChemiDoc™ XRS system with Image Lab™ software, version 6.1 and a 50 bp DNA Ladder™ molecular weight marker (Fermentas^®^, Thermo Fisher Scientific, Waltham, MA, USA).

### 2.4. Acquisition and Identification of Clove Essential Oil (S. aromaticum)

The clove essential oil (EO) was purchased from the company Quinari^®^ (100% pure, ANVISA n° 25351.183090/2017-50, Brazil). According to the manufacturer, clove oil sourced from Indonesia was extracted, with Chemical Abstract Service (CAS) registration numbers 8000-34-8 and 84961-50-2. Leaves and buds were used, and the extraction method was steam distillation. The oil was stored in an amber bottle, protected from light, at room temperature. Qualitatively and quantitatively analyzed at the Chromatography Laboratory of the Chemistry Department, Federal University of Minas Gerais (UFMG, Belo Horizonte, Brazil). The qualitative analysis was performed using gas chromatography coupled with mass spectrometry (GC-MS) on a Shimadzu GCMS-QP2010 ULTRA system. The equipment was operated under the following conditions: Capillary column Restek (30 m × 0.25 mm × 0.25 µm) with oven temperature programmed from 50 °C (0 min), increasing at 3 °C/min until reaching 220 °C; injector temperature at 220 °C with split mode (1:50); GC-MS interface temperature at 230 °C; MS detector (electron impact at 70 eV) at 230 °C; carrier gas: helium at a flow rate of 3 mL/min; injection volume: 1 µL. Data acquisition was performed using the GCMS Solution software (Shimadzu, version 4.0).

The quantitative analysis were performed using High-Resolution Gas Chromatography with Flame Ionization Detection (GC-FID), employing an HP 7820A gas chromatograph (Agilent, Santa Clara, CA, USA) under the following operational conditions: capillary column Rxi-1MS Restek (30 m × 0.25 mm × 0.25 μm); initial oven temperature at 50 °C held for 3 min, ramped at 3 °C/min until reaching 220 °C; injector temperature at 220 °C with split mode (1:50); FID detector temperature at 230 °C; carrier gas: hydrogen at 4 mL/min; injection volume: 1 μL. Data acquisition was performed using the Agilent OpenLab software (version 2.4).

### 2.5. In Vitro Antimicrobial Evaluation of Clove Essential Oil (S. aromaticum)

The antimicrobial activity of the essential oil (EO) of *S. aromaticum* was evaluated through the determination of the minimum inhibitory concentration (MIC) using the broth microdilution technique performed in 96-well microplates. Additionally, the minimum bactericidal concentration (MBC) was determined by plating the wells onto agar plates to verify the bactericidal capacity of the EO. Six (06) methicillin-resistant *S. pseudintermedius* (MRSP) isolates and six (06) methicillin-susceptible *S. pseudintermedius* (MSSP) isolates were evaluated. Dilutions were prepared following a modified protocol established by Freire et al. [40]. The essential oil (EO) was diluted in sterile distilled water to obtain a concentration of 20% or 218.4 mg/mL, which was considered the initial stock solution. To prepare this concentration, 0.5 mL of the essential oil, 0.05 mL of Tween 80, and 4.5 mL of sterile distilled water were added into a sterile glass tube, and then the mixture was vortexed for 5 min using a vortex mixer.

For the MIC determination, the initial stock solution of the essential oil was serially diluted using the broth microdilution method in 96-well microplates, following the protocol described by Aligiannis et al. [41]. Briefly, 100 μL of Mueller–Hinton broth was dispensed into each well using a multichannel pipette, followed by the addition of 100 μL of the essential oil emulsion, resulting in an initial concentration of 10% (109.2 mg/mL) in the first well of the dilution series. Subsequent concentrations were obtained by serial dilution of the product within the microdilution plate, starting from the initial concentration of 10% (109.2 mg/mL—well A1) down to 0.0045% (0.05 mg/mL—well A12), through the transfer of 100 μL from each well to the next. For the wells in column 12, 100 μL of the content was removed to equalize the total volume in each well. Subsequently, 10 μL of the microorganism suspension (1 × 10^8^ CFU/mL) was added to all wells, except in the column designated as the sterility control. The plates were then incubated at 37 °C for 24 h and subsequently analyzed using a spectrophotometer at 625 nm. Methicillin was used as the positive control, and Mueller–Hinton broth as the negative control. For quality control, the standard strain *S. aureus* ATCC 25,923 was employed. All tests were performed in triplicate.

The minimum bactericidal concentration (MBC) was determined by plating 10 μL aliquots of the dilutions corresponding to the MIC, the two immediately preceding, and the two immediately succeeding wells from the microdilution plates onto Mueller-Hinton agar. After plating, the Petri dishes were incubated at 37 °C for 24 h. The MBC was defined as the lowest concentration that prevented visible bacterial growth (complete inhibition). Concentrations at which fewer than three colony-forming units (CFU) were observed were considered bacteriostatic.

## 3. Results

A total of 35 *S. pseudintermedius* isolates were analyzed, obtained from otic swabs of 30 dogs diagnosed with external otitis. Of these 30 dogs, 8 (26.6%) were male and 22 (73.4%) were female. The age distribution of the animals included in the study was as follows: 4 dogs under 1 year old (13.33%), 14 dogs between 1 and 5 years old (46.66%), 7 dogs between 6 and 10 years old (23.33%), and 5 dogs older than 10 years (16.33%). Out of the 35 isolates, 33 (94.28%) showed resistance to at least one tested antimicrobial agent. No isolate exhibited resistance to all antimicrobials tested. The results of antimicrobial susceptibility testing are shown in Table 1.

The highest resistance rates were observed for penicillin, ampicillin, sulfadiazine + trimethoprim, erythromycin, and clindamycin (>50%), with penicillin showing the highest resistance rate (85.71%). The lowest resistance rate was observed for imipenem (2.85%). Other significant resistance rates, ranging from 20% to 40%, were noted for oxacillin, vancomycin, amoxicillin + clavulanic acid, cephalexin, ciprofloxacin, chloramphenicol, doxycycline, enrofloxacin, marbofloxacin, neomycin, polymyxin B, and rifampicin.

The antimicrobial resistance indices (MAR) ranged from 0 to 0.9. Considering this index, the mean MAR value of the studied samples was 0.37. In the present study, 68.57% (24/35) of the isolates were identified as multidrug-resistant. Only two isolates were sensitive to all tested antimicrobials. Regarding the MCAR index, the mean value among the samples was 0.46, ranging from 0 to 0.92. A total of 74.28% (26/35) of the bacterial isolates exhibited an MCAR index greater than 0.25, indicating resistance to three or more classes of antimicrobials and thus classified as multidrug-resistant.

Of the 35 isolates evaluated, 34 (97.14%) harbored at least one of the investigated genes, with 12 (34.28%) presenting both *mecA* and *blaZ* genes simultaneously. Methicillin-resistant *S. pseudintermedius* (MRSP) was detected in 34.28% (12/35) of the isolates by PCR, evidenced by the presence of the *mecA* gene. Among these 12 isolates, 2 (16.66%) were resistant to both oxacillin and cefoxitin in the agar disk diffusion test, 3 (25%) were resistant only to oxacillin, 2 (16.66%) were resistant only to cefoxitin, and 5 (41.66%) showed no resistance to either antimicrobial. Regarding the *blaZ* gene, it was detected in 97.14% (34/35) of the isolates, with only 4 (11.76%) of these 34 isolates being susceptible to penicillin in the agar disk diffusion test.

In the chromatographic analysis of clove essential oil (*S. aromaticum*), the major compounds identified were eugenol (89.8%), β-caryophyllene (7.8%), humulene (1.3%), caryophyllene oxide (0.3%), eugenyl acetate (0.2%), β-selinene (0.1%), and elemol (0.1%). The clove essential oil exhibited antimicrobial activity against both MSSP and MRSP isolates, effectively inhibiting *S. pseudintermedius* at the same concentration (Table 2).

According to the results obtained by the broth microdilution technique, the bacteriostatic activity of *S. aromaticum* essential oil varied among the isolates. The lowest minimum inhibitory concentration (MIC) recorded was 0.21 mg/mL, while the highest was 13.65 mg/mL. The overall mean MIC for all isolates (both MSSP and MRSP) was 6.40 mg/mL. Among the MSSP isolates, the mean MIC was 3.74 mg/mL, and for the MRSP isolates, it was 7.90 mg/mL. The minimum bactericidal concentration (MBC) results were consistent with the MIC values.

## 4. Discussion

External otitis is one of the most commonly observed conditions in dogs [42]. Although this study found a higher percentage of affected females, there is no definitive consensus on sex predisposition for otopathies. However, a higher number of females with external otitis was also reported in a previous study [42]. External otitis can occur at any age, and the age range of affected animals varies considerably. The result found in this study, with a higher frequency in young animals between 1 and 5 years of age, is similar to that reported in the literature [43,44,45].

The high prevalence of antimicrobial resistance observed in the *S. pseudintermedius* isolates (94.28%) is consistent with recent studies that highlight the emergence of multidrug-resistant strains in canine otic infections [46,47]. The high resistance rates to penicillin (85.71%) and ampicillin (65.71%) may be attributed to the presence of the *blaZ* gene, detected in 97.14% of the isolates. This gene encodes a β-lactamase that hydrolyzes the β-lactam ring, conferring resistance to penicillins and first-generation cephalosporins. In the study by Morais et al. [48], 85.2% of *S. pseudintermedius* isolates resistant to β-lactams from dogs with otitis externa harbored the *blaZ* gene, supporting the findings of the present study. β-lactams account for approximately 70% of antimicrobial use in the veterinary sector, according to various clinical studies [49,50,51].

Resistance to erythromycin (65.71%) and clindamycin (51.43%) suggests the possible presence of erm genes, which confer cross-resistance to macrolides, lincosamides, and streptogramins [52,53]. A study by Morais et al. [48] identified *erm* genes in 93% of *S. pseudintermedius* isolates resistant to erythromycin, reinforcing the significance of these resistance mechanisms.

Although vancomycin is primarily used in cases of infections caused by methicillin-resistant *S. aureus* (MRSA) in humans, in this study resistance to this drug was observed in 37.14% of the isolates, a rate considered high, especially given the absence of resistance reported by Feng et al. [54] and Lopes et al. [55], and similar to the findings of Rana et al. [56] and González-Domínguez et al. [57], despite the limited use of vancomycin in veterinary medicine [49,50,51]. The low resistance to imipenem (2.3%) was a significant finding in this study and is supported by the results of Cruz et al. [58], confirming its strong antimicrobial activity and effectiveness against MRSP. Among the antibiotics commonly used in the treatment of canine otitis externa and available in commercial otological formulations, only gentamicin and tobramycin showed favorable susceptibility results against MRSP isolates of *S. pseudintermedius* (>75%).

In this study, resistance to oxacillin by disk diffusion was observed in 28.57% (10/35) of the isolates. However, PCR analysis revealed that only 5 of these strains actually carried the *mecA* gene, while another 7 harbored the gene but were classified as susceptible in the phenotypic test. Considering both methods, 14.28% of the isolates were confirmed as MRSP. The detection of the *mecA* gene in 34.28% of the isolates, along with resistance to oxacillin and cefoxitin, confirms the presence of MRSP. The discrepancy between *mecA* gene detection and phenotypic resistance can be explained by gene mutations or variable expression of resistance [59]. The presence of MRSP is particularly concerning, as it limits therapeutic options and is associated with persistent infections [60]. The discrepancy between genotypic (mecA) and phenotypic methicillin resistance can be partly explained by the occurrence of false-negative or false-positive results in disk diffusion tests. False negatives may arise due to heterogeneous expression of mecA or suboptimal test conditions that do not favor phenotypic expression of resistance. On the other hand, false positives may be related to alternative resistance mechanisms or technical factors that simulate resistance even in the absence of the gene [59,60,61].

Although the *mecA* gene is the most commonly associated gene with methicillin resistance in *S. pseudintermedius*, some studies have shown that another gene, *mecC*, may also be responsible for this resistance profile [2]. This could explain the absence of *mecA* in some of the oxacillin-resistant strains in this study, as well as the fact that some isolates are likely not expressing the resistance gene in vitro. Therefore, antimicrobial monitoring studies in animals carrying these strains are needed to better elucidate the behavior of these bacteria in vivo. It is also worth noting that the accurate detection of MRSP by phenotypic methods is subject to variation due to factors such as inoculum size, incubation time, temperature, pH, and salt concentration. Therefore, the confirmation of MRSP strains through molecular methods is essential [62].

Multidrug resistance was observed in 68.57% of the isolates, with 74.28% exhibiting resistance to three or more classes of antimicrobials (MCAR index > 0.25). These findings are consistent with recent studies [56,63,64] reporting multidrug resistance in over 70% of *S. pseudintermedius* isolates from dogs with otitis and other skin diseases. The average MAR index (0.37) and MCAR index (0.46) underscore the need for strategies to control and monitor antimicrobial resistance in veterinary clinics. Resistance to broad-spectrum antimicrobials such as ciprofloxacin (42.85%) and enrofloxacin (40%) is particularly concerning, as these drugs are frequently used in empirical therapy. Studies highlight that the indiscriminate use of fluoroquinolones in veterinary medicine has contributed to the rise in resistance to these agents [51].

Considering that the improper use of antimicrobials has led to the emergence of multiple resistance in pathogenic microorganisms, there is a need to reduce this indiscriminate use. Thus, natural treatment alternatives are being explored. Among the plants of interest is clove (*S. aromaticum*). Regarding its therapeutic applications, authors identify eugenol, the most abundant component, as responsible for its pharmaceutical use [19]. Although many authors attribute the antimicrobial properties of *S. aromaticum* to eugenol, other compounds have been identified and quantified as constituents of the ethanolic extract, including β-caryophyllene and eugenyl acetate [65].

The chromatographic analysis of the essential oil of clove (*S. aromaticum*) revealed a predominant composition of eugenol (89.8%), followed by β-caryophyllene (7.8%) and humulene (1.3%). This composition is particularly relevant due to the well-documented antimicrobial properties of eugenol, which act through multiple mechanisms, including destabilization of bacterial cell membranes, inhibition of essential enzymes such as ATPases and Krebs cycle enzymes, and suppression of biofilm formation [19,25,66,67,68,69,70,71].

The results demonstrated significant inhibitory activity against both types of *S. pseudintermedius*; methicillin-susceptible strains (MSSP) exhibited MICs ranging from 0.2132 mg/mL (0.0195%) to 6.825 mg/mL (0.625%), while methicillin-resistant strains (MRSP) showed MIC values between 3.4125 mg/mL (0.3125%) and 13.65 mg/mL (1.25%). These results are clinically relevant for several reasons. Firstly, the comparable efficacy against both MRSP and MSSP strains (with an average MIC difference of 3.74 mg/mL vs. 7.9 mg/mL) indicates that β-lactam resistance mechanisms mediated by the *mecA* gene do not confer protection against the components of the essential oil. This observation aligns with previous studies demonstrating the sustained activity of eugenol against MRSA [70,71,72,73,74].

A particularly interesting aspect is the absence of significant cross-resistance. Although two MRSP isolates were not inhibited at the tested concentrations, longitudinal studies indicate that *S. pseudintermedius* develops resistance to eugenol more slowly compared to conventional antibiotics [73,74].

Eugenol (89.8% of the essential oil) exerts a destabilizing effect on the bacterial cell membrane, leading to pore formation and bacterial lysis. This mechanism explains the effective inhibition observed in both MSSP and MRSP strains, even those resistant to β-lactams (*blaZ* and *mecA*). Previous studies have shown that eugenol reduces the expression of *agr* genes (virulence regulators), even at sub-inhibitory concentrations. This effect may contribute to decreased bacterial pathogenicity, supporting the findings of this study, in which the essential oil inhibited 100% of the isolates, including highly resistant MRSP strains. At concentrations of 0.5 × MIC, clove essential oil reduces biofilm formation in MRSP by up to 78% [25,75,76]. This effect is crucial in the context of canine otitis, where bacterial biofilms are common and hinder conventional treatment [73,74,75,76,77,78]; however, the effect of the EO on biofilm was not evaluated in this study.

The clinical implications of these results are significant. The essential oil presents several advantages over conventional antimicrobials: a broad spectrum of action, with simultaneous activity against bacteria and fungi commonly involved in otitis; low cytotoxicity, with in vivo studies demonstrating safety at concentrations ≤2%; an adjunct anti-inflammatory effect mediated by β-caryophyllene, which acts as a CB2 receptor agonist, contributing to edema reduction [79].

However, some limitations must be considered: the volatility and oxidation sensitivity of eugenol require special formulations (such as encapsulation) to ensure stability, and the natural variability in the oil’s composition according to geographic origin demands rigorous standardization processes [80,81].

β-Caryophyllene, a sesquiterpene, exhibits antimicrobial and anti-inflammatory properties and has been shown to enhance the activity of phenolic compounds by increasing cell membrane permeability in bacteria, thereby facilitating greater eugenol uptake [79]. Additionally, eugenyl acetate, an ester derivative of eugenol, possesses moderate antimicrobial effects and may act synergistically by stabilizing or potentiating the activity of eugenol through physicochemical interactions within the essential oil matrix [67]. These minor compounds can modulate the hydrophobicity, volatility, and partitioning behavior of the oil, potentially enhancing its efficacy against bacterial targets.

The antimicrobial activity of clove essential oil (*S. aromaticum*) against *S. pseudintermedius* has not yet been reported. Most studies have tested the antimicrobial action of this essential oil against *S. aureus* and other clinically important pathogenic microorganisms. The MIC found in this study (mean MIC of 6.4 mg/mL or 0.58%) was higher than the MIC values reported by Silvestri et al. [29] for *S. aureus* and by Hill et al. [82] against *Salmonella* Typhimurium. The differences observed in the MIC values may be related to various factors: the type of sample culture, cultivation conditions (incubation time and temperature), culture medium, concentrations of the substances tested, emulsification of the agents used in the oil-water emulsion, and differences in essential oil extraction methods. Additionally, climatic changes can interfere with plant metabolism, as seasonality involves variations in parameters such as temperature and rainfall, which can stimulate the production of certain compounds over others, thereby favoring the synthesis of specific classes of secondary metabolites [80,81].

This study presents promising results regarding the in vitro antibacterial activity of *S. aromaticum* essential oil against *S. pseudintermedius*, including multidrug-resistant strains. However, some limitations should be considered. Firstly, the antimicrobial efficacy was assessed exclusively under in vitro conditions, which may not fully reflect in vivo dynamics, particularly in the complex environment of the canine ear canal. Additionally, the effect of the essential oil on biofilm formation and disruption was not evaluated, despite the known relevance of biofilms in chronic otitis externa. Another limitation concerns the natural variability of essential oil composition, which can affect reproducibility and therapeutic consistency. Future studies should aim to validate these findings in vivo, assess the anti-biofilm potential of clove oil, explore formulation strategies to enhance stability and bioavailability, and investigate potential synergistic effects with conventional antimicrobials.

## 5. Conclusions

This study confirms that *S. pseudintermedius* isolated from dogs with otitis externa shows high rates of antimicrobial resistance, including multidrug resistance and β-lactam resistance mechanisms. The demonstrated in vitro efficacy of *S. aromaticum* essential oil, particularly due to its major constituent eugenol, indicates its potential as an alternative therapeutic option against both oxacillin-susceptible and resistant isolates. These findings highlight the urgent need for prudent antimicrobial use in veterinary practice and support further research into plant-based compounds as complementary or alternative treatments for resistant bacterial infections.

## Figures and Tables

**Table 1:** Antimicrobial susceptibility of 35 *S. pseudintermedius* isolates obtained from dogs with external otitis.

Antimicrobial	Sensitive n° (%)	Intermediate n° (%)	Resistant n° (%)
Amikacin	29 (82.86)	-	06 (17.14)
Ampicillin	12 (34.29)	-	23 (65.71)
Amoxicillin + Clavulanic Acid	27 (77.14)	-	08 (22.86)
Cephalexin	24 (68.57)	01 (2.85)	10 (28.57)
Cefoxitin	29 (82.86)	-	06 (17.14)
Ciprofloxacin	18 (51.43)	02 (5.71)	15 (42.85)
Clindamycin	14 (40.00)	03 (11.42)	18 (51.43)
Chloramphenicol	21 (60.00)	10 (28.57)	04 (11.42)
Doxycycline	17 (48.57)	03 (8.57)	15 (42.85)
Enrofloxacin	19 (54.28)	02 (5.71)	14 (40.00)
Erythromycin	12 (34.28)	-	23 (65.71)
Gentamicin	27 (77.14)	01 (2.85)	07 (20.00)
Imipenem	34 (97.14)	-	01 (2.85)
Marbofloxacin	21 (60.00)	-	14 (40.00)
Neomycin	20 (57.14)	03 (8.57)	12 (34.28)
Oxacillin	25 (71.42)	-	10 (28.57)
Penicillin	05 (14.28)	-	30 (85.71)
Polymyxin B	19 (54.28)	04 (11.42)	12 (34.28)
Rifampicin	20 (57.14)	01 (2.85)	14 (40.00)
Sulfadiazine + Trimethoprim	11 (31.42)	-	24 (65.57)
Tobramycin	28 (80.00)	02 (5.71)	05 (14.28)
Vancomycin	22 (62.85)	-	13 (37.14)

**Table 2:** Minimum inhibitory concentration (MIC) of *S. aromaticum* essential oil and methicillin antibiotic against *S. pseudintermedius*.

Bacteria Analyzed	Essential Oil *Syzygium aromaticum* n° (mg/mL)	Methicillin n° (mg/mL)
*Staphylococcus pseudintermedius* (MSSP)	01 (0.21) 01 (1.79) 04 (6.82)	01 (5×10^−4^) 01 (2 × 10^−3^) 03 (4 × 10^−3^) 01 (8 × 10^−3^)
*Staphylococcus pseudintermedius* (MRSP)	02 (3.41) 02 (6.82) 02 (13.65)	01 (2 × 10^−3^) 01 (4 × 10^−3^) 01 (8 × 10^−3^) 01 (1.28 × 10^−1^) 02 (NI)

MSSP: Methicillin-susceptible *S. pseudintermedius*; MRSP: Methicillin-resistant *S. pseudintermedius*. n°: Number of isolates that exhibited the respective MIC value; NI: Not inhibited at the tested concentrations.

## Data Availability

The original contributions presented in the study are included in the article; further inquiries can be directed to the corresponding author.

## Abbreviations

The following abbreviations are used in this manuscript:

MIC	Minimum Inhibitory Concentration
MBC	Minimum Bactericidal Concentration
SIG	*Staphylococcus intermedius* Group
MRSP	Methicillin Resistance *Staphylococcus pseudintermedius*
MSSP	Methicillin-susceptible *Staphylococcus pseudintermedius*
EO	Essential Oil
MAR	Multiple Antimicrobial Resistance
MCAR	Multiple-Class Antimicrobial Resistance
UFMT	Federal University of Mato Grosso
UFMG	Federal University of Minas Gerais
PCR	Polymerase Chain Reaction
GC-MS	Gas Chromatography Coupled With Mass Spectrometry
GC-FID	Gas Chromatography with Flame Ionization Detection
CFU	Colony-Forming Units
MRSA	Methicillin-Resistant *Staphylococcus aureus*

## References

[1] Beck K., Waisglass S., Dick H., Weese J. (2012). Prevalence of meticillin-resistant *Staphylococcus pseudintermedius* (MRSP) from skin and carriage sites of dogs after treatment of their meticillin-resistant or meticillin-sensitive staphylococcal pyoderma. Vet. Microbiol..

[2] Kjellman E., Slettemea J., Small H., Sunde M. (2015). Methicillin-resistant *Staphylococcus pseudintermedius* (MRSP) from healthy dogs in Norway—Occurrence, genotypes and comparison to clinical MRSP. Microbiologyopen.

[3] Weese J.S., van Duijkeren E. (2010). Methicillin-resistant *Staphylococcus aureus* and *Staphylococcus pseudintermedius* in veterinary medicine. Vet. Microbiol..

[4] Lynch S.A., Helbig K.J. (2021). The complex diseases of *Staphylococcus pseudintermedius* in canines: Where to next?. Vet. Sci..

[5] Borjesson S., Gomez-Sanz E., Ekstrom K., Torres C., Gronlund U. (2015). *Staphylococcus pseudintermedius* can be misdiagnosed as *Staphylococcus aureus* in humans with dog bite wounds. Eur. J. Clin. Microbiol. Infect. Dis..

[6] Robb A.R., Wright E.D., Foster A.M.E., Walker R., Malone C. (2017). Skin infection caused by a novel strain of *Staphylococcus pseudintermedius* in a Siberian husky dog owner. JMM Case Rep..

[7] Somayaji R., Priyantha M.A., Rubin J.E., Church D. (2016). Human infections due to *Staphylococcus pseudintermedius*, an emerging zoonosis of canine origin: Report of 24 cases. Diagn. Microbiol. Infect. Dis..

[8] Moses I.B., Santos F.F., Gales A.C. (2023). Human colonization and infection by *Staphylococcus pseudintermedius*: An emerging and underestimated zoonotic pathogen. Microorganisms.

[9] Blondeau L.D., Deneer H., Rubin J.E., Kanthan R., Sanche S.E., Hamula C.L., Blondeau J.M. (2023). Zoonotic *Staphylococcus pseudintermedius*: An underestimated human pathogen?. Future Microbiol..

[10] Guimarães L., Teixeira I.M., da Silva I.T., Antunes M., Pesset C., Fonseca C., Penna B. (2023). Epidemiologic case investigation on the zoonotic transmission of Methicillin-resistant *Staphylococcus pseudintermedius* among dogs and their owners. J. Infect. Public Health.

[11] Bannoehr J., Guardabassi L. (2012). *Staphylococcus pseudintermedius* in the dog: Taxonomy, diagnostics, ecology, epidemiology and pathogenicity. Vet. Dermatol..

[12] Krapf M., Müller E., Reissig A., Slickers P., Braun S.D., Monecke S. (2019). Molecular characterisation of methicillin-resistant *Staphylococcus pseudintermedius* from dogs and the description of their SCC*mec* elements. Vet. Microbiol..

[13] Nocera F.P., De Martino L. (2024). Methicillin-resistant *Staphylococcus pseudintermedius*: Epidemiological changes, antibiotic resistance, and alternative therapeutic strategies. Vet. Res. Commun..

[14] Alibayov B., Baba-Moussa L., Sina H., Zdeňková K., Demnerová K. (2014). *Staphylococcus aureus* mobile genetic elements. Mol. Biol. Rep..

[15] Peacock S.J., Paterson G.K. (2015). Mechanisms of Methicillin Resistance in *Staphylococcus aureus*. Annu. Rev. Biochem..

[16] Perreten V., Kadlec K., Schwarz S., Andersson U.G., Finn M., Greko C., Moodley A., Kania S.A., Frank L.A., Bemis D.A. (2010). Clonal spread of methicillin-resistant *Staphylococcus pseudintermedius* in Europe and North America: An international multicentre study. J. Antimicrob. Chemother..

[17] Papich M.G. (2012). Selection of antibiotics for meticillin-resistant *Staphylococcus pseudintermedius*: Time to revisit some old drugs. Vet. Microbiol..

[18] Sienkiewicz M., Denys P., Kowalczyk E. (2011). Antibacterial and immunostimulatory effect of essential oils. J. Investig. Allergol. Clin. Immunol..

[19] Marchese A., Barbieri R., Coppo E., Orhan I.E., Daglia M., Nabavi S.F., Ajami M. (2017). Antimicrobial activity of eugenol and essential oils containing eugenol: A mechanistic viewpoint. Crit. Rev. Microbiol..

[20] Nada H.G., Mohsen R., Zaki E.M., Aly A.A. (2022). Evaluation of chemical composition, antioxidant, antibiofilm and antibacterial potency of essential oil extracted from gamma irradiated clove (*Eugenia caryophyllata*). Food Meas. Charact..

[21] Grafakou M.E., Barda C., Karikas G.A., Skaltsa H. (2022). Hypericum Essential Oils—Composition and Bioactivities: An Update (2012–2022). Molecules.

[22] European Medicines Agency (EMA) (2011). Assessment Report on Syzygium aromaticum (L.) Merill et L.M. Perry, Flos and Syzygium aromaticum (L.) Merill et L.M. Perry, Floris Aetheroleum. Committee on Herbal Medicinal Products (HMPC).

[23] Affonso R.S., Rennó M.N., Slana G.B.C.A., Franca T.C.C. (2012). Aspectos Químicos e Biológicos do Óleo Essencial de Cravo da Índia. Rev. Virtual Quim..

[24] Da Silva A.A., Bergamo L., De Camargo L.P., Fernandes C., Mussato D., Canazart D., De Abreu Filho B.A. (2014). Atividade microbiológica de óleos essenciais obtidos por arraste a vapor. UNINGÁ Rev..

[25] Sateriale D., Forgione G., De Cristofaro G.A., Facchiano S., Boscaino F., Pagliuca C., Pagliarulo C. (2022). Towards Green Strategies of Food Security: Antibacterial synergy of essential oils from *Thymus vulgaris* and *Syzygium aromaticum* to inhibit *Escherichia coli* and *Staphylococcus aureus* pathogenic food isolates. Microorganisms.

[26] Beraldo C., Silva D.N., Scanavacca J., Doyaman J.T., Júnior A.F., Moritz C.M.F. (2013). Eficiência de óleos essenciais de canela e cravo-da-índia como sanitizantes na indústria de alimentos. Pesq. Agropec. Trop..

[27] Mostaqim S., Saha S.K., Hani U., Paul S.K., Sharmin M., Basak S., Shahabuddin M.S. (2019). Antibacterial activities of clove (*Syzygium aromaticum*) extracts against three food borne pathogens: *Staphylococcus aureus*, *Escherichia coli* and *Pseudomonas aeruginosa*. Mymensingh Med. J..

[28] Zeshan M.Q., Ashraf M., Omer M.O., Anjum A.A., Ali M.A., Najeeb M., Majeed J. (2023). Antimicrobial activity of essential oils of *Curcuma longa* and *Syzygium aromaticum* against multiple drug-resistant pathogenic bacteria. Trop. Biomed..

[29] Silvestri J.D.F., Paroul N., Czyewski E., Lerin L., Rotava I., Cansian R.L., Mossi A., Toniazzo G., de Oliveira D., Treichel H. (2010). Perfil da composição química e atividades antibacteriana e antioxidante do óleo essencial do cravo-da-índia (*Eugenia caryophyllata* Thunb.). Rev. Ceres.

[30] Quinn P.J., Carter M.E., Markey B., Carter G.R. (1994). Clinical Veterinary Microbiology.

[31] Green R.M., Sambrook J. (2012). Molecular Cloning: A Laboratory Manual.

[32] Sasaki T., Tsubakishita S., Tanaka Y., Sakusabe A., Ohtsuka M., Hirotaki S., Hiramatsu K. (2010). Multiplex-PCR method for species identification of coagulase-positive staphylococci. J. Clin. Microbiol..

[33] CLSI (2013). Performance Standards for Antimicrobial Disk and Dilution Susceptibility Tests for Bacteria Isolated from Animals.

[34] CLSI (2015). Performance Standards for Antimicrobial Susceptibility Testing.

[35] Papich M.G. (2013). Antibiotic treatment of resistant infections in small animals. Vet. Clin. N. Am. Small Anim. Pract..

[36] Krumperman P.H. (1983). Multiple antibiotic resistance indexing of *Escherichia coli* to identify high-risk source of fecal contamination of foods. Appl. Environ. Microbiol..

[37] Ngoi S.T., Thong K.L. (2013). Molecular characterization showed limited genetic diversity among *Salmonella* Enteritidis isolated from humans and animals in Malaysia. Diagn. Microbiol. Infect. Dis..

[38] Choi S.M., Kim S.H., Kim H.J., Lee D.G., Choi J.H. (2003). Multiplex PCR for the detection of genes encoding aminoglycoside modifying enzymes and methicillin resistance among *Staphylococcus* species. J. Korean Med. Sci..

[39] Martineau F., Picard J.F., Lansac N. (2000). Correlation between the resistance genotype determined by multiplex PCR assays and the antibiotic susceptibility patterns of *Staphylococcus aureus* and *Staphylococcus epidermidis*. Antimicrob. Agents Chemother..

[40] Freire I.C.M., Pérez A.L.A.L., Cardoso A.M.R. (2014). Atividade antibacteriana de óleos essenciais sobre *Streptococcus mutans* e *Staphylococcus aureus*. Rev. Bras. Plantas Med..

[41] Aligiannis N., Kalpoutzakis E., Mitaku S. (2001). Composition and antimicrobial activity of the essential oils of two *Origanum* species. J. Agric. Food Chem..

[42] Martins E.A., Momesso C.S., Nardo C.D.D. (2011). Estudo clínico e microbiológico de otite externa de cães atendidos em hospital veterinário do noroeste paulista. Acta Vet. Bras..

[43] Fernandez G., Barboza G., Villalobos A. (2006). Isolation and identification of microorganisms present in 53 dogs suffering otitis externa. Rev. Cient..

[44] Zur G., Lifshitz B., Bdolah-Abram T. (2011). The association between the signalment, common causes of canine otitis externa and pathogens. J. Small Anim. Pract..

[45] Terziev G., Borissov I. (2018). Prevalence of ear diseases in dogs—A retrospective 5-year clinical study. Bulg. J. Vet. Med..

[46] Lee G.Y., Yang S.J. (2020). Comparative assessment of genotypic and phenotypic correlates of *Staphylococcus pseudintermedius* strains isolated from dogs with otitis externa and healthy dogs. Comp. Immunol. Microbiol. Infect. Dis..

[47] Lee G.Y., Lee S.I., Park J.H., Kim S.D., Kim G.B., Yang S.J. (2023). Detection and characterization of potential virulence determinants in *Staphylococcus pseudintermedius* and *S. schleiferi* strains isolated from canine otitis externa in Korea. J. Vet. Sci..

[48] Morais C., Costa S.S., Leal M., Ramos B., Andrade M., Ferreira C., Couto I. (2023). Genetic diversity and antimicrobial resistance profiles of *Staphylococcus pseudintermedius* associated with skin and soft-tissue infections in companion animals in Lisbon, Portugal. Front. Microbiol..

[49] Hur B.A., Hardefeldt L.Y., Verspoor K.M., Baldwin T., Gilkerson J.R. (2020). Describing the antimicrobial usage patterns of companion animal veterinary practices; free text analysis of more than 4.4 million consultation records. PLoS ONE.

[50] Chirollo C., Nocera F.P., Piantedosi D., Fatone G., Della Valle G., De Martino L., Cortese L. (2021). Data on before and after the traceability system of Veterinary antimicrobial prescriptions in small animals at the University Veterinary Teaching Hospital of Naples. Animals.

[51] Glavind A.S., Kruse A.B., Nielsen L.R., Stege H. (2022). Monitoring antimicrobial usage in companion animals: Exploring the use of the Danish VetStat database. Acta Vet. Scand..

[52] Saribas Z., Tunckanat F., Pinar A. (2006). Prevalence of *erm* genes encoding macrolide-lincosamide-streptogramin (MLS) resistance among clinical isolates of *Staphylococcus aureus* in a Turkish university hospital. Clin. Microbiol. Infect..

[53] Thakuri D.R., Pokhrel A., Amatya R., Bashyal N.S., Neupane M., KC S., Khanal S. (2023). Distribution of *MecA* and *Erm* Genes among Methicillin-resistant *Staphylococcus aureus* with Inducible Resistance to Clindamycin. J. Nepal Health Res. Counc..

[54] Feng Y., Tian W., Lin D. (2012). Prevalence and characterization of methicillin-resistant *Staphylococcus pseudintermedius* in pets from South China. Vet. Microbiol..

[55] Lopes G.V., Guerra P.R., Machado V. (2015). Methicillin-resistant *Staphylococcus pseudintermedius* clonal groups isolated from canine pyoderma in Brazil. Acta Sci. Vet..

[56] Rana E.A., Islam M.Z., Das T., Dutta A., Ahad A., Biswas P.K., Barua H. (2022). Prevalence of coagulase-positive methicillin-resistant *Staphylococcus aureus* and *Staphylococcus pseudintermedius* in dogs in Bangladesh. Vet. Med. Sci..

[57] González-Domínguez M.S., Carvajal H.D., Calle-Echeverri D.A., Chinchilla-Cárdenas D. (2020). Molecular Detection and Characterization of the *mecA* and *nuc* Genes from *Staphylococcus* Species (*S. aureus*, *S. pseudintermedius*, and *S. schleiferi*) Isolated from Dogs Suffering Superficial Pyoderma and Their Antimicrobial Resistance Profiles. Front. Vet. Sci..

[58] Cruz A.R., Paes A.C., Siqueira A.K. (2012). Perfil de sensibilidade de bactérias patogênicas isoladas de cães frente a antimicrobianos. Vet. Zootec..

[59] Kadlec K., Weiß S., Wendlandt S., Schwarz S., Tonpitak W. (2016). Characterization of canine and feline methicillin-resistant *Staphylococcus pseudintermedius* (MRSP) from Thailand. Vet. Microbiol..

[60] Viegas F.M., Santana J.A., Silva B.A., Xavier R.G.C., Bonisson C.T., Câmara J.L.S., Silva R.O.S. (2022). Occurrence and characterization of methicillin-resistant *Staphylococcus* spp. in diseased dogs in Brazil. PLoS ONE.

[61] Becker K., van Alen S., Idelevich E.A., Schleimer N., Seggewiß J., Mellmann A., Kaspar U., Peters G. (2018). Plasmid-Encoded Transferable mecB-Mediated Methicillin Resistance in *Staphylococcus aureus*. Emerg. Infect. Dis..

[62] Bonjean M., Hodille E., Dumitrescu O. (2016). Disk diffusion testing for the detection of methicillin-resistant staphylococci: Does moxalactam improve upon cefoxitin?. J. Clin. Microbiol..

[63] Prior C.D., Moodley A., Karama M., Malahlela M.N., Leisewitz A. (2022). Prevalence of methicillin resistance in *Staphylococcus pseudintermedius* isolates from dogs with skin and ear infections in South Africa. J. S. Afr. Vet. Assoc..

[64] Naziri Z., Majlesi M. (2023). Comparison of the prevalence, antibiotic resistance patterns, and biofilm formation ability of methicillin-resistant *Staphylococcus pseudintermedius* in healthy dogs and dogs with skin infections. Vet. Res. Commun..

[65] Santin J.R., Lemos M., Klein-Júnior L.C. (2011). Gastroprotective activity of essential oil of the *Syzygium aromaticum* and its major component eugenol in different animal models. Naunyn Schmiedebergs Arch. Pharmacol..

[66] Alma M.K., Ertas M., Nitz S., Kollmannsberger H. (2007). Chemical composition and content of essential oil from the bud of cultivated Turkish clove (*Syzygium aromaticum*, L.). BioResources.

[67] Chaieb K., Hajlaoui H., Zmantar T. (2007). The chemical composition and biological activity of clove essential oil, *Eugenia caryophyllata* (*Syzygium aromaticum*, L. Myrtaceae): A short review. Phytother. Res..

[68] Gomes P.R.B., Filho V.E.M., Rabelo W.F. (2018). Caracterização química e citotoxicidade do óleo essencial do cravo-da-índia (*Syzygium aromaticum*). Rev. Colomb. Cienc. Quím. Farm..

[69] Radunz M., da Trindade M.L.M., Camargo T.M. (2018). Antimicrobial and Antioxidant Activity of Unencapsulated and Encapsulated Clove (*Syzygium aromaticum*, L.) Essential Oil. Food Chem..

[70] Aelenei P., Miron A., Trifan A., Bujor A., Gille E., Aprotosoaie A.C. (2016). Essential oils and their components as modulators of antibiotic activity against gram-negative bacteria. Medicines.

[71] Raikwar G., Kumar D., Mohan S., Dahiya P. (2024). Synergistic Potential of Essential oils with Antibiotics for Antimicrobial Resistance with Emphasis on Mechanism of Action: A Review. Biocatal. Agric. Biotechnol..

[72] Nascimento G.G., Locatelli J., Freitas P.C., Silva G.L. (2000). Antibacterial activity of plant extracts and phytochemicals on antibiotic-resistant bacteria. Braz. J. Microbiol..

[73] Purkait S., Bhattacharya A., Bag A., Chattopadhyay R.R. (2020). Evaluation of antibiofilm efficacy of essential oil components β-caryophyllene, cinnamaldehyde and eugenol alone and in combination against biofilm formation and preformed biofilms of *Listeria monocytogenes* and *Salmonella typhimurium*. Lett. Appl. Microbiol..

[74] Albuquerque V.D.Q., Soares M.J.C., Matos M.N.C., Cavalcante R.M.B., Guerrero J.A.P., Rodrigues T.H.S., Carneiro V.A. (2021). Anti-staphylococcal activity of *Cinnamomum zeylanicum* essential oil against planktonic and biofilm cells isolated from canine otological infections. Antibiotics.

[75] Nada H., Khatab R., Hashem A., Elhifnawi H., Ashraf A. (2022). In vitro enhancement of ciprofloxacin, tobramycin, and nystatin activity by irradiated aqueous garlic extract against multidrug-resistant pathogens causing otitis. Arab. J. Nucl. Sci. Appl..

[76] Mustafa E.A., Hashem A.E.G., Elhifnawi H.N., Nada H.G., Khattab R.A. (2021). One-pot biosynthesis of silver nanoparticles with potential antimicrobial and antibiofilm efficiency against otitis media–causing pathogens. J. Clin. Microbiol. Infect. Dis..

[77] Duarte M.C.T., Leme C., Figueira G.M. (2007). Effects of essential oils from medicinal plants used in Brazil against EPEC and ETEC *Escherichia coli*. Rev. Bras. Plantas Med..

[78] Stefanetti V., Passamonti F., Rampacci E. (2024). Antimicrobial strategies proposed for the treatment of *S. pseudintermedius* and other dermato-pathogenic *Staphylococcus* spp. in companion animals: A narrative review. Vet. Sci..

[79] Fidyt K., Fiedorowicz A., Strządała L., Szumny A. (2016). β-caryophyllene and β-caryophyllene oxide—Natural compounds of anticancer and analgesic properties. Cancer Med..

[80] Rio J.L., Recio M.C. (2005). Medicinal plants and antimicrobial activity. J. Ethnopharmacol..

[81] Liñán-Atero R., Aghababaei F., García S.R., Hasiri Z., Ziogkas D., Moreno A., Hadidi M. (2024). Clove essential oil: Chemical profile, biological activities, encapsulation strategies, and food applications. Antioxidants.

[82] Hill L.E., Gomes C., Taylor T.M. (2012). Characterization of β-cyclodextrin inclusion complexes containing essential oils (trans-cinnamaldehyde, eugenol, cinnamon bark, and clove bud extracts) for antimicrobial delivery applications. Food Sci. Technol..

